# Structure of the first representative of Pfam family PF09410 (DUF2006) reveals a structural signature of the calycin superfamily that suggests a role in lipid metabolism

**DOI:** 10.1107/S1744309109037749

**Published:** 2009-12-08

**Authors:** Hsiu-Ju Chiu, Constantina Bakolitsa, Arne Skerra, Andrei Lomize, Dennis Carlton, Mitchell D. Miller, S. Sri Krishna, Polat Abdubek, Tamara Astakhova, Herbert L. Axelrod, Thomas Clayton, Marc C. Deller, Lian Duan, Julie Feuerhelm, Joanna C. Grant, Slawomir K. Grzechnik, Gye Won Han, Lukasz Jaroszewski, Kevin K. Jin, Heath E. Klock, Mark W. Knuth, Piotr Kozbial, Abhinav Kumar, David Marciano, Daniel McMullan, Andrew T. Morse, Edward Nigoghossian, Linda Okach, Jessica Paulsen, Ron Reyes, Christopher L. Rife, Henry van den Bedem, Dana Weekes, Qingping Xu, Keith O. Hodgson, John Wooley, Marc-André Elsliger, Ashley M. Deacon, Adam Godzik, Scott A. Lesley, Ian A. Wilson

**Affiliations:** a Joint Center for Structural Genomics, http://www.jcsg.org, USA; b Stanford Synchrotron Radiation Lightsource, SLAC National Accelerator Laboratory, Menlo Park, CA, USA; c Program on Bioinformatics and Systems Biology, Burnham Institute for Medical Research, La Jolla, CA, USA; d Lehrstuhl für Biologische Chemie, Technische Universität München, 85350 Freizing-Weihenstephan, Germany; e Department of Medicinal Chemistry, College of Pharmacy, University of Michigan, Ann Arbor, MI 48109-1065, USA; f Department of Molecular Biology, The Scripps Research Institute, La Jolla, CA, USA; g Center for Research in Biological Systems, University of California, San Diego, La Jolla, CA, USA; h Protein Sciences Department, Genomics Institute of the Novartis Research Foundation, San Diego, CA, USA; i Photon Science, SLAC National Accelerator Laboratory, Menlo Park, CA, USA

**Keywords:** structural genomics, domains of unknown function, calycin, lipocalin, fatty-acid binding proteins

## Abstract

The first structural representative of the domain of unknown function DUF2006 family, also known as Pfam family PF09410, comprises a lipocalin-like fold with domain duplication. The finding of the calycin signature in the N-­terminal domain, combined with remote sequence similarity to two other protein families (PF07143 and PF08622) implicated in isoprenoid metabolism and the oxidative stress response, support an involvement in lipid metabolism. Clusters of conserved residues that interact with ligand mimetics suggest that the binding and regulation sites map to the N-terminal domain and to the interdomain interface, respectively.

## Abstract 2

NE1406, the first structural representative of PF09410, reveals a lipocalin-like fold with features that suggest involvement in lipid metabolism. In addition, NE1406 provides potential structural templates for two other protein families (PF07143 and PF08622).

## 1. Introduction

In an effort to extend the structural coverage of proteins for which the biological function is unknown and cannot be deduced by homology (domains of unknown function; DUFs), targets were selected from Pfam protein family PF09410 (DUF2006). Here, we report the crystal structure of NE1406, the first structural representation of this family, which was determined using the semiautomated high-throughput pipeline of the Joint Center for Structural Genomics (JCSG; Lesley *et al.*, 2002 ▶) as part of the NIGMS Protein Structure Initiative (PSI). The *NE1406* gene of *Nitrosomonas europaea*, an obligate chemolithoautotroph, encodes a protein with a molecular weight of 40.1 kDa (residues 1–356) and a calculated isoelectric point of 5.0.

## 2. Materials and methods

### 2.1. Protein production and crystallization

Clones were generated using the polymerase incomplete primer extension (PIPE) cloning method (Klock *et al.*, 2008 ▶). The gene encoding NE1406 (GenBank NP_841447, gi|30249377, Swiss-Prot Q82US3) was amplified by polymerase chain reaction (PCR) from *N. europaea* strain ATCC 19718 genomic DNA using *PfuTurbo* DNA polymerase (Stratagene) and I-PIPE (Insert) primers (forward primer 5′-ctgtacttccagggcATGCGTTACTTATGGATACTGTTG-3′, reverse primer 5′-aattaagtcgcgttaCATCGATAACGGACGTACG-3′; target sequence in upper case) that included sequences for the predicted 5′ and 3′ ends. The expression vector pSpeedET, which encodes an amino-terminal tobacco etch virus (TEV) protease-cleavable expression and purification tag (MGSDKIHHHHHHENLYFQ/G), was PCR-amplified with V-PIPE (Vector) primers. V-PIPE and I-PIPE PCR products were mixed to anneal the amplified DNA fragments together. *Escherichia coli* GeneHogs (Invitrogen) com­petent cells were transformed with the V-PIPE/I-PIPE mixture and dispensed onto selective LB-agar plates. The cloning junctions were confirmed by DNA sequencing. Using the PIPE method, the part of the gene encoding residues Met1–Pro22 was deleted. Expression was performed in a selenomethionine-containing medium with suppression of normal methionine synthesis. At the end of fermentation, lysozyme was added to the culture to a final concentration of 250 µg ml^−1^ and the cells were harvested and frozen. After one freeze–thaw cycle, the cells were sonicated in lysis buffer [50 m*M* HEPES pH 8.0, 50 m*M* NaCl, 10 m*M* imidazole, 1 m*M* tris(2-­carboxyethyl)phosphine–HCl (TCEP)] and the lysate was clarified by centrifugation at 32 500*g* for 30 min. The soluble fraction was passed over nickel-chelating resin (GE Healthcare) pre-equilibrated with lysis buffer, the resin was washed with wash buffer [50 m*M* HEPES pH 8.0, 300 m*M* NaCl, 40 m*M* imidazole, 10%(*v*/*v*) glycerol, 1 m*M* TCEP] and the protein was eluted with elution buffer [20 m*M* HEPES pH 8.0, 300 m*M* imidazole, 10%(*v*/*v*) glycerol, 1 m*M* TCEP]. The eluate was buffer-exchanged with TEV buffer (20 m*M* HEPES pH 8.0, 200 m*M* NaCl, 40 m*M* imidazole, 1 m*M* TCEP) using a PD-10 column (GE Healthcare) and incubated with 1 mg TEV protease per 15 mg of eluted protein. The protease-treated eluate was run over nickel-chelating resin (GE Healthcare) pre-equilibrated with HEPES crystallization buffer (20 m*M* HEPES pH 8.0, 200 m*M* NaCl, 40 m*M* imidazole, 1 m*M* TCEP) and the resin was washed with the same buffer. The flowthrough and wash fractions were combined and concentrated by centrifugal ultrafiltration (Millipore) to 19.4 mg ml^−1^ for crystallization trials. NE1406 was crystallized using the nanodroplet vapor-diffusion method (Santarsiero *et al.*, 2002 ▶) with standard JCSG crystallization protocols (Lesley *et al.*, 2002 ▶). Sitting drops composed of 200 nl protein mixed with 200 nl crystallization solution were equilibrated against a 50 µl reservoir at 293 K for 50 d prior to harvest. The crystallization reagent consisted of 1.4 *M* ammonium sulfate and 0.1 *M* CHES [2-(*N*-cyclohexylamino)ethanesulfonic acid] pH 9.0. Glycerol was added to the crystal to a final concentration of 10%(*v*/*v*) as a cryoprotectant. Initial screening for diffraction was carried out using the Stanford Automated Mounting system (SAM; http://smb.slac.stanford.edu/facilities/hardware/SAM/UserInfo; Cohen *et al.*, 2002 ▶) at the Stanford Synchrotron Radiation Lightsource (SSRL; Menlo Park, California, USA). Diffraction data from a plate-shaped crystal with approximate dimensions 0.2 × 0.1 × 0.05 mm mounted in a nylon loop were indexed in the orthorhombic space group *P*2_1_2_1_2_1_ (Table 1 ▶). The oligomeric state of NE1406 was determined to be a monomer using a 0.8 × 30 cm Shodex Protein KW-803 column (Thomson Instruments) pre-calibrated with gel-filtration standards (Bio-Rad). Protein concentrations were determined using the Coomassie Plus assay (Pierce).

### 2.2. Data collection, structure solution and refinement

Multiple-wavelength anomalous diffraction (MAD) data were collected at the APS on beamline 23-ID-D at wavelengths corresponding to the inflection (λ_1_), high-energy remote (λ_2_) and peak (λ_3_) points of the Se *K* absorption spectrum. The data sets were collected at 100 K using a MAR Mosaic300 CCD detector (Rayonix). The MAD data were integrated and reduced using *MOSFLM* (Leslie, 1992 ▶) and scaled with the program *SCALA* (Collaborative Computational Project, Number 4, 1994 ▶). Phasing was performed with *SOLVE* (Terwilliger & Berendzen, 1999 ▶), with a mean figure of merit of 0.28 with eight selenium sites (no selenium site was found for the disordered C-terminal SeMet356 for either chain). Density modification with *RESOLVE* (Terwilliger, 2002 ▶) was followed by automated model building with *ARP*/*wARP* (Cohen *et al.*, 2004 ▶). Model completion and refinement were carried out with *Coot* (Emsley & Cowtan, 2004 ▶) and *REFMAC* 5.2 (Winn *et al.*, 2003 ▶) using data set λ_1_. Refinement included experimental phase restraints in the form of Hendrickson–Lattman coefficients from *SOLVE*, NCS restraints (positional weights of 0.5 and 5.0 and thermal weights of 2.0 and 10.0 for the main-chain and side-chain atoms, respectively) and TLS refinement with one group per chain. NCS restraints were applied as two sets: to the N-terminal residues 24–74 and the C-terminal residues 83–351. Data-collection and refinement statistics are summarized in Table 1 ▶.

### 2.3. Validation and deposition

**Figure 1:**
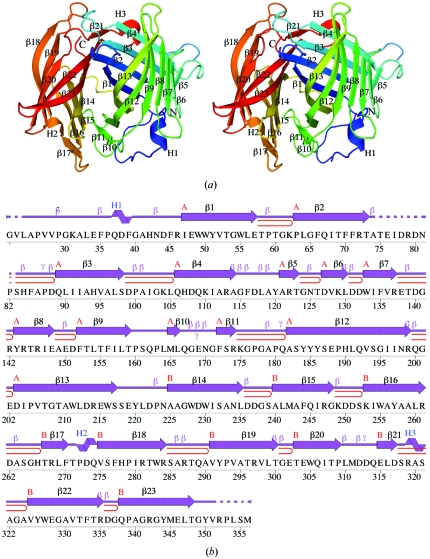
Crystal structure of NE1406 from *N. europaea*. (*a*) Stereo ribbon diagram of the NE1406 monomer (chain *A*) color-coded from the N-terminus (blue) to the C-terminus (red). Helices H1–H3 and β-strands β1–β23 are indicated. (*b*) Diagram showing the secondary-structure elements of NE1406 (chain *A*) superimposed on its sequence. The labeling of secondary-structure elements is in accord with *PDBsum* (http://www.ebi.ac.uk/pdbsum), where α-helices are sequentially labeled (H1, H2, H3 *etc*.), β-strands are labeled (A, B, C *etc*.) according to the β-sheets to which they are assigned, β-turns and γ-turns are designated by Greek letters (β, γ) and β-hairpins by red loops. For NE1406, the 3_10_-helices (H1–H3), β-strands in β-sheets (A and B, comprising strands β1–β13 and β14–β23, respectively ), β-turns (β) and γ-turns (γ) are indicated. Dashed lines indicate sections of sequence in the construct that are not modeled in the structure.

Analysis of the stereochemical quality of the model was accomplished using *AutoDepInputTool* (Yang *et al.*, 2004 ▶), *MolProbity* (Davis *et al.*, 2007 ▶), *SFCHECK* 4.0 (Collaborative Computational Project, Number 4, 1994 ▶) and *WHAT IF* 5.0 (Vriend, 1990 ▶). Protein quaternary structure was analyzed using the *PISA* server (Krissinel & Henrick, 2007 ▶). Fig. 1 ▶(*b*) was adapted from an analysis using *PDBsum* (Laskowski *et al.*, 2005 ▶) and all other figures were prepared with *PyMOL* (DeLano Scientific). Atomic coordinates and experimental structure factors for NE1406 at 2.0 Å resolution have been deposited in the PDB with code 2ich.

## 3. Results and discussion

### 3.1. Overall structure

The crystal structure of a truncated version of NE1406 (Fig. 1 ▶ *a*) was determined to 2.0 Å resolution using the MAD phasing technique. Data-collection, model and refinement statistics are summarized in Table 1 ▶. The final model includes 643 residues in two protein molecules (*A* and *B*), two CHES molecules, three glycerol molecules, one sulfate ion and 394 water molecules in the asymmetric unit. No electron density was observed for Gly0 (from the purification tag), Val23 (the first residue after Gly0), Thr75–Pro82 and Arg352–SeMet356 in chain *A* or for Thr75–Asp80 and Pro353–SeMet356 in chain *B*. The side-chain atoms of Leu24, Arg144, Glu169, Gln200, Asp222 from chain *A* and Leu24, Gln89 and Arg352 from chain *B* were omitted owing to poor electron density. The two chains are nearly identical, with an r.m.s.d. of 0.30 Å over 320 C^α^ atoms (0.60 Å over all 2524 equivalent atoms). The Matthews co­efficient (*V*_M_; Matthews, 1968 ▶) is 2.35 Å^3^ Da^−1^ and the estimated solvent content is 47.3%. The Ramachandran plot produced by *MolProbity* (Davis *et al.*, 2007 ▶) shows that 98 and 100% of the residues are in favored and allowed regions, respectively.

SCOP classifies NE1406 as an all-β protein with an AttH-like fold characterized by two flattened, orthogonally packed, β-barrels of lipocalin-like topology (http://scop.mrc-lmb.cam.ac.uk/scop/data/scop.b.c.bai.b.b.b.html). Lipocalins (PF00061) are an increasingly diverse family of predominantly small, single-domain, secreted proteins exhibiting high affinity and selectivity for hydrophobic molecules. Structurally, lipocalins form a subset of the calycin superfamily, which additionally includes avidins and fatty-acid binding proteins (FABPs) (Flower *et al.*, 1993 ▶; Pfam clan CL0116). Calycins are an example of a superfamily with members sharing structural similarities that cannot be detected at the sequence level. The calycin core fold comprises an eight-stranded calyx-shaped antiparallel β-barrel which opens toward one end, where the binding site is located. In the case of lipocalins and avidins, the core fold is maintained and differences are observed in the loop lengths and compactness of the barrel. In FABPs, the core calycin fold is supplemented by two additional β-strands and two short helices that pack on top of the lipid-binding cavity. In all cases, a short 3_10_-helix caps the barrel at one end, which is also latched by a conserved cation–π interaction involving a tryptophan from the first β-strand and a lysine or arginine residue from the final β-strand of the barrel. Both of these residues additionally form hydrogen bonds to main-chain atoms in the 3_10_-helix (Flower *et al.*, 2000 ▶).

**Figure 2:**
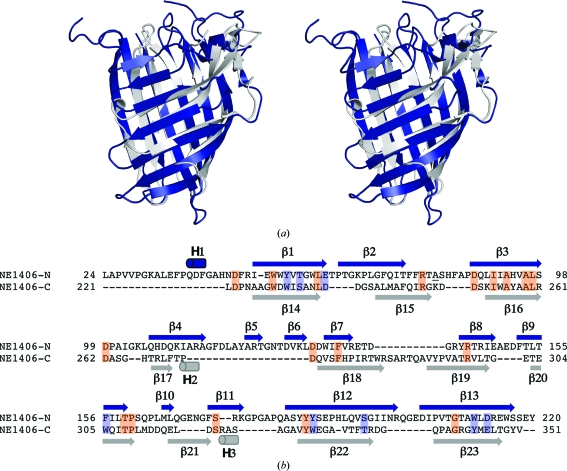
NE1406 exhibits domain duplication. (*a*) Stereo ribbon diagram of the N-terminal domain (residues 24–220, blue) of NE1406 superimposed onto the C-terminal domain (residues 221–352, gray). (*b*) Structure-guided alignment of the N- and C-terminal domains of NE1406. Secondary-structure elements are indicated in blue and gray for the N- and C-terminal domains, respectively. Identical residues are boxed in orange and conservative substitutions in purple. Ala74 is underlined to denote the eight-residue break in the chain between Ala74 and Ser83. The missing region was not modeled owing to poor electron density and is likely to be flexible.

The N-terminal domain of NE1406 (residues 24–220) comprises 13 β-strands arranged in the form of a flattened barrel with a 3_10_-helix (H1 in Fig. 1 ▶) capping the barrel at one end (Fig. 1 ▶ *a*). The C-terminal domain (residues 221–352) is arranged perpendicular to the long axis of the N-terminal barrel and comprises ten β-strands. It can be superimposed on the N-terminal domain with a C^α^ r.m.s.d. of 2.4 Å over 105 residues (Fig. 2 ▶ *a*), suggesting gene duplication, although the sequence identity of only 9% is nonsignificant (Fig. 2 ▶ *b*). Strands β5–β6 are absent from the C-terminal domain, while β11 is replaced by another 3_10_-helix (H3 in Fig. 2 ▶ *b*). The 3_10_-helix cap of the N-terminal barrel is replaced by two longer strands, β18–β19 (in the C-terminal domain), that extend over one end of the barrel (Figs. 1 ▶ *a* and 2 ▶).

### 3.2. Detection of the calycin superfamily signature

A search with *FATCAT* (Ye & Godzik, 2004 ▶) using the entire NE1406 structure gave no significant hits. Individually, the N- and C-­terminal domains both showed structural similarity to a variety of β-barrel proteins, including outer membrane proteins (PDB codes 2erv, 2jmm, 1k24 and 1p4t), avidin-related and streptavidin-related proteins (PDB codes 1avd, 1wbi, 1y52, 2ciq, 2uyw and 1stp), fatty-acid binding proteins (PDB codes 1g5w and 2q9s), nitrophorin (PDB codes 1d2u and 1u17) and a retinoic acid-binding protein (PDB code 1blr). The best score was for the outer membrane protein PagL from *Pseudomonas aeruginosa* (PDB code 2erv), which gave a C^α^ r.m.s.d. of 3.4 Å over 198 residues with a sequence identity of only 3%.

**Figure 3:**
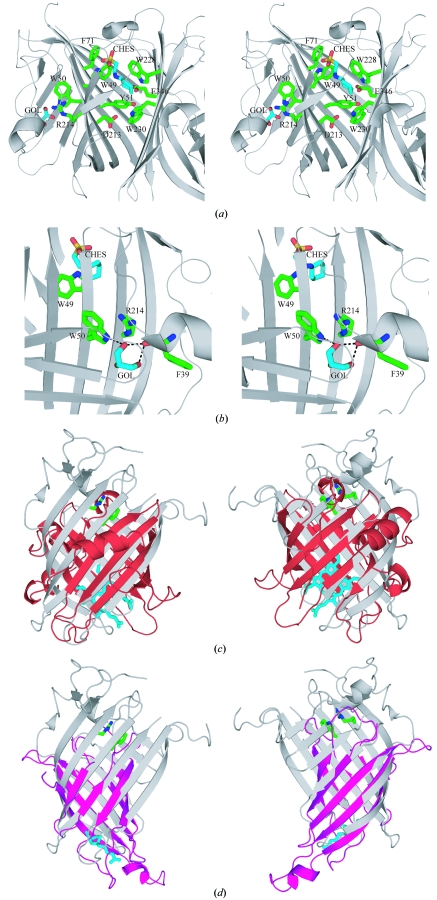
Similarities and differences between NE1406 and the calycin superfamily. (*a*) Stereo ribbon diagram of the binding sites for the two buffer molecules 2-(*N*-cyclohexylamino)ethanesulfonic acid (CHES) and glycerol (GOL). Conserved residues are indicated. (*b*) NE1406 exhibits the calycin-superfamily structural signature. Stereo ribbon diagram of the N-terminal domain of NE1406 showing the stacked arginine and tryptophan residues characteristic of the calycin fold (Flower *et al.*, 2000 ▶). Hydrogen bonds are indicated by dashed lines. A glycerol molecule (cyan) mediates bonding of Trp50 to the 3_10_-helix. (*c*) Ribbon diagrams depicting the front and back view of NE1406 (PDB code 2ich, residues 24–220; gray) superposed with nitrophorin 4 from *Rhodnius prolixus* (PDB code 1d2u, residues 22–205; red. The heme ligand for nitrophorin 4 is colored cyan. (*d*) Ribbon diagrams depicting the front and back view of NE1406 (PDB code 2ich, residues 24–220; grey) superposed with avidin from *Gallus gallus* (PDB code 1avd, residues 3–125; pink). The Trp-Arg signatures are represented as sticks. The biotin ligand for avidin is shown in cyan.

This calycin-family signature in NE1406 (Fig. 3 ▶ *b*) is conserved in the DUF2006 family. In the N-terminal domain of NE1406, the Arg214 side chain from β13 interacts with main-chain residues in both β1 and the N-terminal 3_10_-helix, whereas hydrogen bonding of the Trp50 indole to the 3_10_-helix is mediated *via* a glycerol molecule (Fig. 3 ▶ *b*). Although the calycin signature is absent from the NE1406 C-terminal domain (Fig. 2 ▶), its presence in the N-terminal domain served to direct our analysis towards calycin-superfamily members.

Analysis of the structural superposition of NE1406 with members of the calycin superfamily revealed a number of systematic differences (Figs. 3 ▶ *c* and 3 ▶ *d*). The β-sheets forming the NE1406 β-barrel are both longer and flatter than those in lipocalins, resulting in a narrower opening at the bottom of the barrel where the lipocalin-binding site would reside. The difference is even more pronounced when NE1406 is compared with avidins (PF01382; Fig. 3 ▶ *d*), which have barrels that are more circular and compact than in lipocalins. In this respect, NE1406 resembles FABPs, which also exhibit a barrel that is flatter and more elliptical than in lipocalins. However, NE1406 lacks two additional helices at the top of the barrel that are a characteristic of FABPs. Secondary-structure elements, such as the long C-terminal α-­helix characteristic of most lipocalin-like calycins, *e.g.* nitrophorin (PF02087; Flower *et al.*, 2000 ▶; Skerra, 2000 ▶), are also absent from NE1406. Finally, the calycin signature residues are in different conformations to those typically described for calycins, with Trp50 adopting a different rotamer in NE1406 than in calycins and Arg214 not adopting a fully extended conformation.

### 3.3. Similarities and differences with lipocalins

NE1406 is likely to provide the first structural template for two other protein families. A search with *HHpred* (Soding *et al.*, 2005 ▶) against Pfam gave *E* values of 1.0 × 10^−15^ and 1.5 × 10^−7^ for protein families PF07143 and PF08622, respectively. PF07143 is a prokaryotic family of hydroxyneurosporene synthases that are implicated in carotene metabolism, while PF08622 is a family of fungal proteins that inhibit the generation of reactive oxygen species and promote survival during oxidative stress. The role of isoprenoids in photoprotection in plants (Penuelas & Munne-Bosch, 2005 ▶) and antioxidant defence in other eukaryotes (Tapiero *et al.*, 2004 ▶; Rao & Rao, 2007 ▶) has been well documented. A number of lipocalins, such as apolipoprotein D (ApoD; Sanchez *et al.*, 2006 ▶; Charron *et al.*, 2008 ▶; Eichinger *et al.*, 2007 ▶), neutrophil gelatinase-associated lipocalin (Roudkenar *et al.*, 2008 ▶; Goetz *et al.*, 2002 ▶) and α_1_-microglobulin (Olsson *et al.*, 2008 ▶; Schonfeld & Wojtczak, 2008 ▶), provide protection against oxidative stress by means of isoprenoids such as carotene. Other members of the calycin superfamily, such as avidins (PF01382), are not involved in this response. We therefore searched for other indications that NE1406 might be related to the lipocalin/cytosolic fatty-acid binding protein family (PF00061).

Lipocalins have been likened to antibodies because of the high degree of structural plasticity that their binding sites exhibit, with numerous examples in which structural consolidation occurs upon binding (for a review, see Skerra, 2008 ▶). As a result, the lipocalin fold has been employed in a number of protein-engineering studies (Beste *et al.*, 1999 ▶; Korndorfer *et al.*, 2003 ▶). In the NE1406 crystal structure, the two lipocalin-like barrels lack the large internal cavity that is typical of lipocalins and also the long structurally flexible loops at the open end of the β-barrel (Skerra, 2000 ▶). In fact, only one of the β-­barrel domains of NE1406 harbors a small glycerol molecule from the crystallization solution as a ligand. However, the complete internalization of the glycerol molecule in the NE1406 structure suggests that the N-terminal lipocalin-like barrel might adopt different conformations in the presence of a natural ligand. We therefore propose that this region, which encompasses the calycin signature, acts as a ligand-binding site, the shape and accessibility of which may change with natural ligands.

The ability to form dimers is another feature of the lipocalin family, with ligand presence influencing oligomerization (Grzyb *et al.*, 2006 ▶). Analytical size-exclusion chromatography shows that NE1406 forms a monomer in solution, whereas crystal-packing analysis suggests a dimer with a total buried surface area of 1290 Å^2^ per monomer. While it is possible that dimerization of NE1406 is modulated by ligand binding, the relative orientation of the two protein domains within the polypeptide chain could also be subject to regulation by a second ligand. The two barrels are stabilized in a perpendicular orientation with respect to each other. The mainly aromatic and hydrophobic residues implicated in the interaction with CHES are highly or strictly conserved among DUF2006 homologs, suggesting that the domain interface plays a functional role. As with the glycerol molecule bound within the N-terminal barrel, the CHES molecule is also fully enclosed within NE1406 with no exposure to solvent, suggesting some flexibility at the interdomain interface to accommodate ligands. Ligand binding at the domain interface might act to regulate the shape of the binding cavity within one or both of the β-barrels in a similar manner to the regulation by dimerization observed in lipocalins.

Finally, some lipocalins, such as the bacterial lipocalin (Blc), ApoD and lazarillo, are known to be peripherally anchored to biological membranes, where they are thought to play a role in membrane biogenesis and repair (Bishop, 2000 ▶; Eichinger *et al.*, 2007 ▶). Expressed under conditions known to exert stress on the bacterial envelope, Blc from *E. coli* has a high affinity for lysophospholipids (LPLs), which may also be bound inside the β-barrel and are thought to be involved in cell-envelope LPL transport (Campanacci *et al.*, 2006 ▶). Although the exact mechanisms of transperiplasmic movement of lipids between inner and outer membranes are largely unknown, ATP-binding cassette transporters are involved in this process (Doerrler *et al.*, 2004 ▶).

As expected, a search with *PROFtmb* (Bigelow *et al.*, 2004 ▶) shows that NE1406 is not predicted to be a transmembrane β-barrel (*Z* score 2.9). However, calculations with the program *PPM* (Lomize *et al.*, 2006 ▶) suggest weak peripheral association of the protein with membrane. The ligand-binding cavity of the β-barrel opens towards the membrane surface in the predicted orientation (Supplementary Fig. 1^1^), similar to ApoD (Eichinger *et al.*, 2007 ▶). The membrane-interacting residues of the protein include the exposed hydrophobic Phe85 and a large patch of basic residues (Arg46, Arg113, Lys249, Arg284, Arg287, Arg319 and Arg352).

### 3.4. Genome-context analysis

The genome context (http://string.embl.de) of NE1406 shows a predicted functional association with the lipoprotein-releasing system ATP-binding protein LolD (*lolD*) and co-occurrence with an ATP-binding protein ABC transporter (NE1404). A high degree of con­fidence is predicted for the functional association of many DUF2006 homologs with ATP-dependent ABC transporters, as well as with other transmembrane proteins including Na^+^/H^+^ antiporters, sensor histidine kinases and lipoproteins (*e.g.* LprI precursor in *Mycobacterium tuberculosis*). The systematic presence of ATP-dependent cassettes and lipoproteins is compatible with a role for the DUF2006 family in lipid transport, while the presence of numerous signal transduction genes might indicate expression under specific conditions, such as environmental stress. Further experiments will be required in order to functionally characterize NE1406 and to determine whether it associates with lipids *in vitro* or *in vivo* and whether its transcription is subject to environmental regulation.

The DUF2006 protein family contains over 400 homologs distributed among trypanosomata, fungi, mycobacteria, bacteroidetes, rhizobia, *Vibrio*, spirochaetes, firmicutes and archaea. Given the wide phylogenetic presence of the DUF2006 family, if an experimental connection to lipocalins is determined, this finding would present the first evidence of a lipocalin-related protein in the Archaea domain and would settle the question of whether or not this protein family may have arisen *via* horizontal transfer to eukaryotic cells from the endosymbiotic α-proteobacterial ancestor of the mitochondrion (Bishop, 2000 ▶).

The availability of more DUF2006 sequences and structures might shed light on the evolutionary history of this intriguing protein family. The information presented here, in combination with further bio­chemical and biophysical studies, should yield valuable insights into the functional role of NE1406. Models of NE1406 homologs can be accessed at http://www1.jcsg.org/cgi-bin/models/get_mor.pl?key=2ichA.

Additional information about the protein described in this study is available from *TOPSAN* (Krishna *et al.*, 2010 ▶) at http://www.topsan.org/explore?PDBid=2ich.

## 4. Conclusions

NE1406 adopts a lipocalin-like fold with domain duplication. Analysis based on the calycin-superfamily signature present in the N-­terminal domain reveals a potential binding site, while remote sequence homology and the genome context suggest involvement in isoprenoid metabolism and survival under oxidative stress.

## Supplementary Material

PDB reference: NE1406 from *N. europaea*, 2ich, r2ichsf

Supplementary material file. DOI: 10.1107/S1744309109037749/wd5116sup1.pdf

## Figures and Tables

**Table 1:** Summary of crystal parameters, data-collection and refinement statistics for NE1406 (PDB code 2ich) Values in parentheses are for the highest resolution shell.

	λ_1_ MADSe	λ_2_ MADSe	λ_3_ MADSe
Data collection			
Space group	*P*2_1_2_1_2_1_		
Unit-cell parameters (Å)	*a* = 63.27, *b* = 95.57, *c* = 121.75		
Wavelength (Å)	0.9794	0.9493	0.9792
Resolution range (Å)	29.20–2.00 (2.05–2.00)	29.20–2.00 (2.05–2.00)	29.10–2.00 (2.05–2.00)
No. of observations	178048	177082	176130
No. of unique reflections	49800	49531	49656
Completeness (%)	98.4 (95.9)	97.9 (95.0)	98.4 (95.6)
Mean *I*/σ(*I*)	9.3 (2.1)	9.8 (2.3)	8.8 (2.0)
*R*_merge_ on *I*†	0.117 (0.599)	0.109 (0.535)	0.121 (0.602)
Model and refinement statistics			
Resolution range (Å)	29.2–2.00		
No. of reflections (total)	49646‡		
No. of reflections (test)	2528		
Completeness (%)	98.0		
Data set used in refinement	λ_1_		
Cutoff criterion	|*F*| > 0		
*R*_cryst_§	0.182		
*R*_free_¶	0.232		
Stereochemical parameters			
Restraints (r.m.s.d. observed)			
Bond angles (°)	1.65		
Bond lengths (Å)	0.018		
Average isotropic *B* value (Å^2^)	27.9††		
ESU‡‡ based on *R*_free_ (Å)	0.16		
Protein residues/atoms	643/5142		
Water molecules/ions/other solvent§§	394/1/5		

† *R*_merge_ = 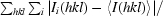 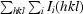.

‡ Typically, the number of unique reflections used in refinement is slightly less that the total number that were integrated and scaled. Reflections are excluded owing to systematic absences, negative intensities and rounding errors in the resolution limits and unit-cell parameters.

§ *R*_cryst_ = 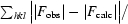 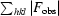, where *F*_calc_ and *F*_obs_ are the calculated and observed structure-factor amplitudes, respectively.

¶ *R*_free_ is the same as *R*_cryst_ but for 5.1% of the total reflections chosen at random and omitted from refinement.

†† This value represents the total *B* that includes TLS and residual *B* components.

‡‡ Estimated overall coordinate error (Collaborative Computational Project, Number 4, 1994 ▶; Cruickshank, 1999 ▶).

§§ Two CHES and three glycerol molecules.

## Footnotes

1 Supplementary material has been deposited in the IUCr electronic archive (Reference: WD5116).
