# Supplementary material for: Structure of the first representative of Pfam family PF09410 (DUF2006) reveals a structural signature of the calycin superfamily that suggests a role in lipid metabolism
Source: Acta Crystallogr Sect F Struct Biol Cryst Commun. 2009 Dec 8;66(Pt 10):1153–9. doi: 10.1107/S1744309109037749 (PMC2954199; doi:10.1107/S1744309109037749)
Supplement: Supplementary file 1 [file f-66-01153-sup1.pdf]

Tina Bakolitsa 9/18/2009
2ich_SN SNTS: 569

Supplementary Material

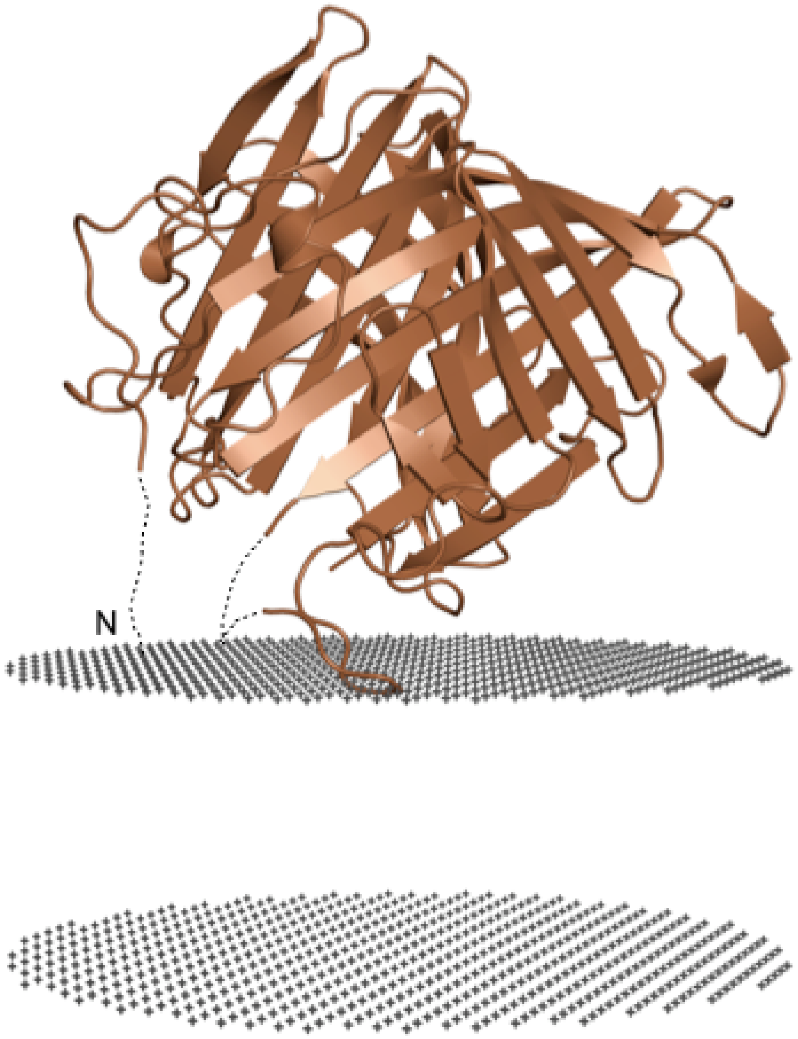

Figure S1. Model of NE1406 interacting with a membrane. The hydrocarbon boundary planes of the lipid bilayer (head groups of lipids go ~10 Å beyond these boundaries) are depicted as hashed disks. Dashed lines indicate the regions not modeled in the protein structure. The predicted N-terminus is indicated.
